# Intra-Abdominal Hypertension and Abdominal Compartment Syndrome in Association with Ruptured Abdominal Aortic Aneurysm in the Endovascular Era: Vigilance Remains Critical

**DOI:** 10.1155/2012/151650

**Published:** 2012-02-21

**Authors:** Matthew C. Bozeman, Charles B. Ross

**Affiliations:** Division of Vascular Surgery and Endovascular Therapeutics, Department of Surgery, University of Louisville, Louisville, KY 40202, USA

## Abstract

Intra-abdominal hypertension (IAH) and abdominal compartment syndrome (ACS) are common complications of ruptured abdominal aortoiliac aneurysms (rAAAs) and other abdominal vascular catastrophes even in the age of endovascular therapy. Morbidity and mortality due to systemic inflammatory response syndrome (SIRS) and multiple organ failure (MOF) are significant. Recognition and management of IAH are key critical care measures which may decrease morbidity and improve survival in these vascular patients. Two strategies have been utilized: expectant management with prompt decompressive laparotomy upon diagnosis of threshold levels of IAH versus prophylactic, delayed abdominal closure based upon clinical parameters at the time of initial repair. Competent management of the abdominal wound with preservation of abdominal domain is also an important component of the care of these patients. In this review, we describe published experience with IAH and ACS complicating abdominal vascular catastrophes, experience with ACS complicating endovascular repair of rAAAs, and techniques for management of the abdominal wound. Vigilance and appropriate management of IAH and ACS remains critically important in decreasing morbidity and optimizing survival following catastrophic intra-abdominal vascular events.

## 1. Introduction

Patients who survive an initial operation for an intra-abdominal catastrophic event, such as a ruptured abdominal aortic aneurysm (rAAA) or other abdominal vascular catastrophes such as complicated mesenteric revascularization, often suffer from severe physiologic derangements. Factors contributing to their critical illness include hemodynamic instability, massive fluid resuscitation, transfusion of blood products, hypothermia, acidosis, and lengthy operations with resultant fluid shifts. ACS is recognized as a major cause of death after treatment for rAAA with short-term mortality rates as much as 5 times greater than observed in patients without ACS even in patients treated by endovascular techniques [1]. Although endovascular repair of rAAA (REVAR) has shown promise in reducing these challenges and improving survival in patients with suitable anatomy [2], a reported 10–20% of patients treated by endovascular techniques still develop ACS [1–6]. Recognition and management of IAH and ACS complicating open or endovascular repairs is critically important in decreasing morbidity and improving survival from rAAA.

Kron et al. [7] in 1984 described ACS and end-organ failure emphasizing oliguria managed successfully with decompressive laparotomy following an initial operation for repair of rAAA. Fietsam et al. [8] first described delayed abdominal closure and subsequent management in 1989. Since that time, further experiences and lessons learned from diagnosing and treating ACS have been reported [1, 4, 9–20].

## 2. The Physiologic Basis of Abdominal Compartment Syndrome

The end result of intra-abdominal hypertension is impaired organ perfusion resulting in MOF. A relationship exists as a continuum between IAH and ACS. IAH is evaluated with a grading system reported in 2006 by the World Conference on Abdominal Compartment Syndrome (Table 1) [21]. IAH is defined as an intra-abdominal pressure (IAP) greater than 12 mmHg in consecutive, standardized measurements with the risk of progression to overt ACS, defined as IAP greater than 20 mmHg or abdominal perfusion pressure less than 60 mmHg with end-organ dysfunction [21], increasing as IAP increases. Early in the disease process, prior to stage III, patients develop oliguria, elevated end-inspiratory pressures, and hypoperfusion due to decreasing cardiac output [22]. With unabated progression of the clinical syndrome, high peak inspiratory pressures with hypercarbia, impaired venous return, marked oliguria to anuria unresponsive to fluid challenge, altered levels of consciousness, and respiratory failure are encountered [23]. At or above an intra-abdominal pressure of 25 mmHg, extensive bowel necrosis, frequently involving the left colon, is observed. Malperfusion of the gut in this clinical continuum may be particularly threatening when vascular operations have altered mesenteric blood supply, that is, coverage of a previously patent inferior mesenteric artery, hypogastric artery, or both.

From the WSACS consensus definitions, 2004 [21], major risk factors for the development of ACS in patients suffering from acute intra-abdominal vascular catastrophe are listed in Table 2. Knowledge of these risk factors combined with calculation of IAP or abdominal perfusion pressure (abdominal perfusion pressure = mean arterial pressure-intra-abdominal pressure) may guide management. The WSACS defined an abdominal perfusion pressure (APP) of 60 mmHg or less to be consistent with poor perfusion and an increased risk for ischemia. Cheatham et al. [24] found the abdominal perfusion pressure (target of 50 mmHg) to be superior to intra-abdominal pressure measurements, arterial pH, urinary output, lactate, and base deficit measurements as an endpoint of resuscitation and a better predictor of survival in patients with IAH and ACS.

## 3. Measuring Intra-Abdominal Pressure

**Figure 1:**
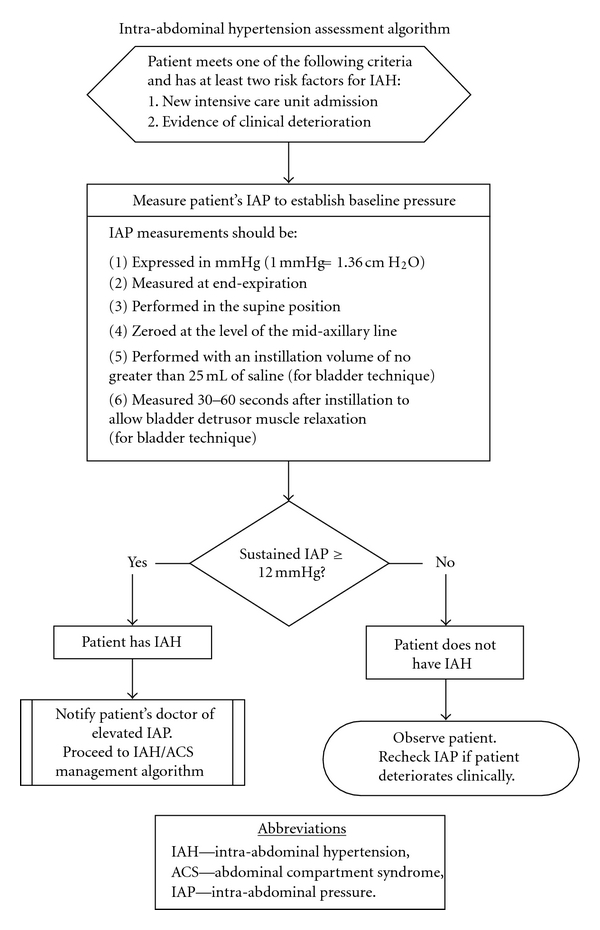
From: Cheatham et al. [25] Intensive Care Med. 2007 Jun; 33(6):951-62.

Various methods have been described for the accurate measurement of intra-abdominal pressure. Puncture of the abdominal wall for pressure measurement carries unacceptable risk, making indirect techniques necessary. Most commonly, intra-cystic pressures are used as a surrogate for intra-abdominal pressure. Djavani Gidlund [4] described inflation of 50 mL of sterile saline into the aspiration port of a Foley catheter, clamping the tube distal to the port, and connecting a 16-gauge needle to a transducer to measure the pressure transmitted across the bladder wall. Because of the potential for spuriously elevated pressures, current protocols specify inflation of the Foley balloon with only 25 mL of saline [25]. The midaxillary line is used as the zero point for the supine patient. Intermittent indirect measurement methods have been described, and Balogh et al. [26] detailed a continuous measurement technique based on a three-way Foley catheter and irrigation. A standardized algorithm was published in 2007 by WSACS assessing IAH [25] (Figure 1).

 Another technique to measure bladder pressure is the Foleymanometer [27] system (Holtech Medical, Copenhagen Denmark), which can be applied to both ICU patients as well as patients in noncritical care settings. This system uses the patient's own urine as the pressure transducing medium. A 50 mL container filled with biofiller that is able to be vented is inserted between the Foley catheter and the drainage bag. During normal drainage, this container fills with urine, but once elevated, 50 mL of urine flows back into the patient's bladder creating a pressure transducing column. The tubing is clear and marked to allow easy reading of the patients' IAP. Malbrain [28] found excellent correlation between this technique and those previously described. 

## 4. Incidence of Abdominal Compartment Syndrome after rAAA

Vascular surgeons have not always recognized the significance of ACS as a determinate of survival in rAAA patients. In a survey of practicing vascular surgeons in Australia reported in 2008, Choi et al. [29] found that only 30% routinely measured intra-abdominal pressure in postoperative rAAA patients and that only 17% would consider prophylactic delayed abdominal wound closure in rAAA patients meeting criteria making them high risk for postoperative development of ACS. It follows that the true incidence of ACS as a contributing factor to MOF and death following repair of rAAA may be underappreciated due, in part, to failure to monitor for its occurrence. Another factor contributing to the under recognition of ACS in these patients is that, prior to 2004, no consensus definition existed to define IAH and ACS and to guide management.

Fietsam et al. [8] reported a retrospective review from 1978–1988 in which 4/104 (5.8%) patients surviving an rAAA repair developed ACS, apparent to the authors even without a standardized definition at that time. Two of these patients were managed with an open abdomen at the conclusion of their repair. Rasmussen et al. [12] from the Mayo Clinic reported a case control series of 223 patients treated for rAAA over a ten-year period. Of 90 patients closed primarily and used as casecontrols in this study, 10 (11%) developed postoperative ACS and were treated with decompressive laparotomy. However, it should be noted that 53 patients in this series were managed with prophylactic delayed closure at the time of initial operation. In 2010, Starnes et al. [30] reported sobering data on the incidence ACS in rAAA patients managed by open repair and their subsequent outcomes. Thirty patients of 151 managed by open repair developed ACS (19%). Mortality in those who developed ACS was 66%.

Djavani Gidlund, et al. [4] published findings on a consecutive series of 29 patients treated by REVAR and monitored postoperatively utilizing IAP monitoring guidelines from the 2004 WSACS consensus. IAH was observed in 16 (55%), with 6 patients (21%) developing IAH >20 mmHg and 3 (10%) of those patients developing ACS. Three (10%) of these patients developed overt ACS. Starnes et al. [30] reported the development of ACS in 2 of 27 (7.4%) patients managed by REVAR with 1 of the 2 patients surviving.

These data confirm that IAH and ACS occur commonly in the setting of rAAA whether managed by open techniques or by REVAR.

## 5. Abdominal Compartment Syndrome in the Age of Endovascular Repair

The advent of endovascular repair of abdominal aortic aneurysms has revolutionized the management of abdominal aortic aneurysms. Although first successfully performed in 1994 by Marin and colleagues [31], widespread experience with the use of endovascular techniques for rAAA (REVAR) has lagged elective procedures, but encouragingly, more and more centers are developing standardized protocols and patient selection criteria to treat rAAA with endovascular grafts [2, 28]. REVAR, in theory, allows the patient to undergo a less physiologically challenging procedure, especially given the multiple comorbidities carried by patients who present with rAAA. Care strategies for patients with rAAA have played a part in the development of REVAR. The term hypotensive hemostasis describes the use of permissive hypotension to limit both the pathologic and iatrogenic resuscitative effects of a catastrophic rupture by limiting the size of the retroperitoneal hematoma and reducing the amount of preoperative transfusions and fluids. Most centers have instituted protocols which tolerate systolic blood pressure (SBP) of <90 to 80 mmHg or even lower depending on neurocognitive function as an indicator of instability, thus limiting the volume of resuscitative fluids and blood products prior to exclusion of the rupture [1, 5, 28, 32]. Supraceliac aortic balloon occlusion is utilized in hemodynamically unstable patients for rapid control of aortic hemorrhage. REVAR has been performed under general, epidural, and local anesthesia with sedation as well. In our center, we favor permissive hypotension as low as a systolic pressure of 80 mmHg with sustained level of consciousness combined with local anesthesia and sedation for REVAR. Whether the broad adoption and implementation of these management strategies prior to definitive aortic repair will decrease the incidence of postoperative IAH and ACS remains to be seen.

Mayer et al. [32] retrospectively reviewed an extensive, single center's experience spanning the decade from 1998–2008 with REVAR. Starting in 2000, this group used REVAR, by protocol, for all rAAA patients with favorable anatomy and accessible iliac arteries. Supraceliac balloon control was utilized in 19/102 (19%) of patients. Hemodynamic instability was defined in three ways: all patients with SBP ≤70 mmHg were termed to be in circulatory shock (31 patients), SBP <50 mmHg in severe shock (14), and persistent shock, that is, impending circulatory collapse SBP <50 mmHg (19) despite adequate resuscitation. Permissive hypotension was utilized with a target blood pressure of 70–90 mmHg. After successful REVAR, the rate of ACS was 20% as defined by intra-abdominal pressure >20 mmHg and signs of organ failure. The 30-day mortality rate in their series was 13%. All patients with ACS were managed by decompressive laparotomy, but the mortality in this cohort was not reported.

Djavani Gilund et al. [4] reported a series of 32 patients with rAAA treated by REVAR. 25% of these patients presented in shock. They used a standard protocol in 29/32 patients in which postoperative IAP measurements were performed every 4 hours with the FoleyManometer [26] system. All patients who presented in shock developed some degree of IAH. Patients with an IAP of >12 mmHg were treated conservatively with neuromuscular blockade, pain relief, diuretics, and colloid resuscitation. Mortality for those that developed ACS was significantly higher (67% versus 13%, *P* = .01), but overall mortality achieved by REVAR was 13%.

A recent study by Veith et al. in 2009 [5] attempted to define the role of endovascular versus open repair via a questionnaire sent to 49 centers reporting experience with treatment of REVAR. Questionnaires returned demonstrated 1037 patients treated with EVAR versus 763 treated by open repair. The responses returned varied concerning ACS. Some routinely monitored IAP, others opened the abdomen when pressures reached 25 mmHg without signs of organ failure, others used WSACS guidelines throughout. Overall 30-day mortality rates for rAAA treated in these centers were 21% for REVAR and 36% for open repair (*P* < 0.0001). The role IAH and ACS played in the outcomes of these patients was not delineated. The proportion of REVAR patients, treated in the surveyed centers, in whom ACS was recognized and treated by decompressive laparotomy, hematoma evacuation, or open abdominal techniques averaged 12.2 ± 8.3%.

In summary, ACS remains a major issue facing vascular surgical and critical care teams regardless of the repair chosen. REVAR has been demonstrated to be a successful and promising strategy to treat rAAA, but vigilance for the development of ACS and its subsequent management remains an essential component contributing to favorable outcomes.

## 6. When to Decompress?

The gold standard for treatment of ACS is decompressive laparotomy. However, escalating care to an open abdomen state carries with it real concerns for the patient. Strategies exist for management of IAH which has not progressed to ACS. An IAP >12 mmHg should signal the critical care team that physiologic derangements are evolving and progression to ACS is possible. Neuromuscular blockade for ventilated patients is often a useful treatment measure, especially early in the course of the disease process. Relaxation of the abdominal and thoracic musculature can allow increased intra-abdominal domain and reduce the intra-abdominal pressure. Neuromuscular blockade should always be combined with sedation and analgesia. Other treatment strategies such as adequate pain control, including the use of parenteral medications and/or thoracic epidural anesthesia, conservative crystalloid use, correcting positive fluid balance with careful use hypertonic colloid solutions, diuresis and goal directed resuscitation, and the use of vasopressors to support an abdominal perfusion pressure ≥60 mmHg offer temporizing strategies for patients with IAH but not fully developed ACS [25, 26].

These conservative measures are no longer appropriate once ACS has been identified. An IAP of >30 mmHg, especially, can signal impending cardiovascular collapse and urgent decompression is mandatory [25]. A full midline laparotomy should be utilized once ACS is recognized. The immediate decrease in IAP can exacerbate hypotension in the periprocedural time period, and the need for further resuscitation should be anticipated.

Percutaneous catheter decompression (PCD) is receiving increasing attention as an alternative to decompressive laparotomy in the treatment of patients with ACS [33]. Patients with rAAA treated by REVAR may have free intra-abdominal blood and fluid amenable to percutaneous drainage. However, active hemorrhage and coagulopathy may contraindicate PCD; hence, the use of this technique following REVAR requires caution and further study. Continuous monitoring of IAP and parameters of organ function is necessary if PCD is employed because PCD may not be successful in relieving ACS in all cases. Cheatham and Safcsak [33] recently reported successful avoidance of decompressive laparotomy in 25 of 31 patients with ACS treated by PCD. A small cohort of these patients had had vascular procedures, but no further information was provided. This technique is particularly attractive as an alternative to open abdominal management in patients who have required aortic replacement, whether by standard or endovascular techniques, but further clinical experience is required to define its role in the vascular setting.

## 7. Prophylactic Delayed Abdominal Wound Closure versus Decompressive Laparotomy

Prophylaxis against the development of IAH and ACS at the end of the index procedure may be achieved by using open abdomen management strategies, that is, delayed abdominal closure. Intra-abdominal bladder pressure readings may be falsely reassuring in a patient with an abdomen open at the end of a procedure, and in such cases, clinical factors may be used to decide whether or not to primarily close the fascia. Patient presentation (degree of hypotension, acidosis, hypothermia, resuscitation requirement), operative details (length >4 hours, intra-operative resuscitation, blood loss, etc.), the amount of bowel and abdominal wall edema, abdominal tension, and its effect on peak airway pressures at attempted closure suggest that IAH and ACS are likely to occur in the postoperative period.

Rasmussen et al. [12] used delayed closure of the abdominal wall with a mesh bridge in such situations. Severe base deficit (−14 versus −7), increased fluid resuscitation (4.0 L/hr versus 2.7 L/hr), and hypothermia (32°C versus 35°C) were used as surrogates to predict the need for mesh closure. When compared to a subset of patients requiring decompressive laparotomy due to the development of ACS, those patients closed with mesh at the initial operation had lower MOF scores (*P* < 0.05), a lower mortality rate (51% versus 70%), and were more likely to survive their MOF (70% versus 11%, *P* < 0.05). This data supports the use of prophylactic delayed abdominal closure in those at risk for postoperative ACS.

The decision to perform a decompressive laparotomy with conversion to an open abdomen or to prophylactically delay abdominal wound closure is not taken lightly by vascular surgical teams. Graft infection has long been a dreaded complication of aortic replacement, and fears of increased risk of graft infection with an open abdomen may cause hesitation in definitive management of ACS by vascular surgeons. Multiple studies (Table 3), however, have shown aortic graft infection in the setting of open abdominal management to be a very rare complication [7–20]. Ross et al. [17] reported a series of 23 patients from both a community and academic hospital setting where prophylactic delayed abdominal closure was employed based on clinical risk factors after operation for intra-abdominal catastrophe, most of which were rAAA. Eighteen of 23 survived. Abdominal wound closure for rAAA patients, all of whom were repaired by open techniques, was achieved at a mean of 4 days. Graft infection was not observed in this series of patients with a mean followup of 53 months.

A more recent study by Acosta et al. [20] evaluating the fascial closure rate of patients treated with an open abdomen utilizing vacuum and mesh-mediated traction techniques did identify a single patient with an aortic stent graft infection. In their study, 111 patients were treated with the combined vacuum and mesh-mediated traction technique (VAWCM), and of these 45 patients were treated secondary to vascular disease. The authors reported a mortality of 29.7%, median time to closure of 14 days, a 76.6% primary fascial closure rate overall in their intention-to-treat analysis, as well as an 89% fascial closure rate in per-protocol analysis. Factors associated with failure of closure included development of intestinal fistula and >14 days of open abdomen treatment. A single patient of 30 (3%) reported with repair of a rAAA developed an aortic graft infection. However, no other information specifically pertaining to this single patient was presented in their analysis.

Risk factors for development of ACS in association with REVAR reported by Mehta et al. [1] include aortic balloon occlusion, massive transfusion, coagulopathy, and conversion to an aorto-unilateral device. In 6 of 30 patients, in this group's initial experience, treated by REVAR, the diagnosis was made in 4 at the time of the index procedure. Decompressive laparotomy was immediately performed and 2 of the 4 survived. Death was observed in 2 patients in whom the diagnosis and decompressive laparotomy were delayed. Based on this experience, on-table decompressive laparotomy was recommended for patients with abdominal distention, signs of organ failure, and risk factors for ACS regardless of intraoperative bladder pressure measurements. No clear recommendations have otherwise been established to guide the timing of decompressive laparotomy in the setting of REVAR. One note of caution, however, relates to the phenomenon of decompression bleeding with decompressive laparotomy in this setting. Marin et al. [31] reported one death due to this phenomenon and now take measures to close the site of rupture at the time of decompressive laparotomy. Coagulopathy may contribute to this phenomenon.

## 8. Management of the Abdominal Wound

Many different techniques have been described for maintaining abdominal domain and eventually achieving closure, either by permanent mesh, absorbable mesh, tissue transfer, or closing fascia primarily [14]. Our preference is to use a negative pressure, vacuum-pack system either with or without a mesh fascial bridge [17]. Advantages of using mesh sutured to the fascia, with bowel protected by underlying plastic sheeting and towels, include maintenance of abdominal domain, avoidance of dehiscence, and a theoretical avoidance of possible fistula formation when applying vacuum suction. Patients with a negative pressure dressing incorporating a mesh bridge can even be extubated and managed off the ventilator prior to definitive closure. At the time of definitive closure, the mesh is removed and fascia sewn together. Opponents of this technique argue that suturing mesh to fascia is unnecessary and may cause weakening or even necrosis of the fascia, subsequently decreasing fascial integrity at the time of definitive closure. When mesh is not used, we create a negative pressure dressing by placing a soft, fenestrated plastic sheet as a barrier between the bowel and abdominal wall. This sheet both prevents adherence of bowel to the abdominal wall and allows escape of fluids. The sheet is then covered by a soft towel with 2 to 4 large, flat drains placed on the surface of the towel. Two layers of an iodine-impregnated drape are placed over the top to form an airtight seal. The drains are then placed to wall suction. When mesh is used, the system is largely unchanged, with the polypropylene mesh placed over the plastic barrier and towel and affixed to the fascia with a 0-Prolene running suture. Drains were then placed on top of the mesh and covered with towels followed by iodine-impregnated occlusive dressings. The appliance was then placed to wall suction as mentioned previously. Early in the senior author's experience, the mesh bridge combined with negative pressure was used. However, most cases presently encountered in our center are managed by negative pressure alone, either with the previously mentioned surgeon created vacuum pack or the commercially available VAC system (vacuum-assisted closure, KCI, San Antonio, Tex) Advantages of the commercially available system include a standardized method of placing the system, ability to adjust the negative pressure applied, and having portable systems available. However, the VAC system is more expensive in the short term than the previously described surgeon-created system with similar rates of complications [34].

Trips back to the operating room for “washouts” and examination of abdominal contents are kept to a minimum. It is the senior author's preference that the integrity of the occlusive dressing be vigorously maintained and that returns for laparotomy be limited, when possible, to a single trip for definitive fascial closure. Achievement of negative fluid balance, normal peak inspiratory pressures, absence of transfusion requirement, and a pliable abdomen guide the timing of operative returns for definitive closure.

Björck et al. [35] have devised a classification scheme (Table 4) designed to aid in the description of the patient's course, as well as to improve reporting of OA management and standardize some of the clinical guidelines for treatment of this heterogeneous patient population. This scheme, while providing a clear system for classification and broad guidelines for management of the patient with an open abdomen, has yet to be examined in prospectively randomized studies.

Use of the open abdomen is an essential component of damage control surgery as practiced by modern trauma and acute care surgery teams. Smith and colleagues [36] have reported use of directed peritoneal resuscitation with 2.5% glucose-based peritoneal dialysis solution (Delflex, Fresenius, USA) at a rate of 1.5 mL/kg/hour in a negative pressure dressing until closure. Using this protocol, they achieved earlier definitive fascial closure as compared to standard management, that is, 4.35 ± 1.6 versus 7.05 ± 3.31 days, *P* < 0.003, respectively. The same team has presented data from animal models demonstrating favorable modulation of the inflammatory cascade by this technique [37]. The utility and risk associated with this management technique in the setting of rAAA has yet to be defined but holds promise.

## 9. Conclusions

Management of patients with intra-abdominal catastrophic vascular events such as rAAA and complicated mesenteric revascularization for ischemic bowel will continue to challenge vascular surgical and critical care teams even in the endovascular era. IAH and ACS are predictable complications which must be promptly recognized and managed to prevent excessive morbidity and mortality. Correct identification of patients at risk, standardized monitoring of IAP, prompt recognition of the disease in patients with closed abdomens, and selective use of prophylactic delayed abdominal closure can optimize outcomes.

## Figures and Tables

**Table 1:** Grading of intra-abdominal hypertension (WSACS consensus definitions, 2004).

Grade I	IAP 12–25 mmHg
Grade II	IAP 16–20 mmHg
Grade III	IAP 21–25 mmHg
Grade IV	IAP > 25

IAP = Intra-abdominal pressure.

**Table 2:** Established risk factors for IAH/ACS in acute abdominal vascular catastrophe.

*Adapted from WSACS consensus definitions, 2004 [21]*
Hemoperitoneum and retroperitoneal hematoma
Massive fluid resuscitation (>5 L colloid or crystalloid/24 h)
Polytransfusion (>10 U packed red blood/24 h)
Coagulopathy (platelets <55,000/mm3 or activated partial
thromboplastin time two times normal or higher or prothrombin
time <50% *or *international standardized ratio *>*1.5)
Hypothermia (core temperature <33°C)
Acidosis (pH < 7.2)
Lengthy cross-clamp time or balloon-occlusion time
Lengthy operative times

**Table 3:** Experience with open abdominal management following open repair of rAAA (adapted and updated from Ross et al. [17]).

Reference	Number of patients/ number reopened for ACS	Technique	Time to closure (days)	Survival (%)	Graft infection	Mean followup
Kron et al. [7]	4/4			100	None	
Fietsam et al. [8]	6/4	Marlex mesh bridge		50	None	
Akers et al. [9]	6 (1/6 TAAA)	Silicone rubber sheet		50	None	
Oelschlager et al. [10]	8	Plastic sheet (6), Skin closure (2)	12 (median)	50	None	
Ciresi et al. [11]	9	Gore-tex bridge	7.9 ± 3.2	78	None	
Rasmussen et al. [12]	45/10	Mesh (Plastic 69%, PTFE 13%, other 18%) Sewn to fascia (84%), sewn to skin (16%)	2–7 (range)	44		Actuarial 32% survival (95% CI 19–54%) to 5 years
Foy et al. [13]	21/4	Plastic sheet			None	
Barker et al. [14]	22/3	Primary fascial closure (14), skin graft/mesh (2)	4 ± 3.3	59.1	None	
Kushimoto et al. [15]	5	Soft tissue flap	4 (median)	80	None	
Petersson et al. [16]	7	Mesh bridge	32 (median)	100	None	9 months (median)
Ross et al. [17]	23	All vacuum packed, mesh bridge (9), towel to fascia (4), no fascial fixation (10)	5.3 ± 6 (2 to 29) 4 in rAAA patients (2 to 7)	78	None	53 ± 24 months (13 to 107 months)
Seternes et al. [18]	9/7	Vacuum packed with mesh sewn to fascia	10.5 (median), 6–19 (range)	66	None	17 months
Morisaki et al. [19]	3	Vacuum packed with plastic bag to fascia	6.3	100	None	
Acosta et al. [20]	30*	Vacuum packed with mesh traction closure	Undefined*	70	1 aortic stent graft	

*30 patients in the Acosta et al. [20] series were treated for rAAA. Details specific to these patients were, otherwise, unreported with the exception of one aortic stent graft infection.

**Table 4:** Proposed classification of the open abdomen. Adapted from Björck et al. [35].

Grade	Description
1A	Clean OA without adherence between bowel and
	abdominal wall or fixity (lateralization of the
	abdominal wall)
1B	Contaminated OA without adherence/fixity
2A	Clean OA developing adherence/fixity
2B	Contaminated OA developing adherence/fixity
3	OA complicated by fistula formation
4	Frozen OA with adherent/fixed bowel; unable to
	close surgically; with or without fistula
